# Arterial Function in Healthy Pregnant Women vs. Non-Pregnant Women—A 10-Year Study

**DOI:** 10.3390/diagnostics10060374

**Published:** 2020-06-05

**Authors:** Vladiana Turi, Simona Dragan, Mircea Iurciuc, Lavinia Moleriu, Simona Bungau, Delia Mirela Tit, Daniela-Oana Toader, Camelia Cristina Diaconu, Tapan Behl, Izabella Petre

**Affiliations:** 1 Department of Cardiology, “Victor Babeş” University of Medicine and Pharmacy, 2 Eftimie Murgu Sq., 300041 Timisoara, Romania; turi.vladiana@umft.ro (V.T.); simona.dragan@umft.ro (S.D.); mirceaiurciuc@gmail.com (M.I.); 2 Department III Functional Sciences, Faculty of Medicine, “Victor Babes” University of Medicine and Pharmacy, 2 Eftimie Murgu Sq., 300041 Timisoara, Romania; moleriu.lavinia@umft.ro; 3 Department of Pharmacy, Faculty of Medicine and Pharmacy, University of Oradea, 29 N. Jiga St., 410028 Oradea, Romania; mirela_tit@yahoo.com; 4 Department 13, Faculty of Medicine, “Carol Davila” University of Medicine and Pharmacy, 050474 Bucharest, Romania; oana.toader@yahoo.com; 5 “Alessandrescu–Rusescu” National Institute for Mother and Child Health, Bucharest, Polizu Clinical Hospital, 011062 Bucharest, Romania; 6 Department 5, Faculty of Medicine, “Carol Davila” University of Medicine and Pharmacy, 050474 Bucharest, Romania; drcameliadiaconu@gmail.com; 7 Department of Internal Medicine, Clinical Emergency Hospital of Bucharest, 014461 Bucharest, Romania; 8 Chitkara College of Pharmacy, Chitkara University, Punjab 140401, India; tapanbehl31@gmail.com; 9 Department XII of Obstetrics and Gynaecology, “Victor Babeş” University of Medicine and Pharmacy, 2 Eftimie Murgu Sq., 300041 Timisoara, Romania; dr.petreizabella@yahoo.com

**Keywords:** arterial stiffness, pulse wave velocity, augmentation index, pregnancy, cardiovascular risk

## Abstract

Introduction. Maternal age for the first pregnancy is increasing and so, the prevalence of cardiovascular risk factors in pregnancy is also increasing. Heart disease is the main reason for maternal death during pregnancy in developed countries. Arterial stiffness is an independent risk factor for atherosclerosis and a predictor of cardiovascular morbidity and mortality. The most widespread parameters for detecting subclinical atherosclerosis are augmentation index (AIx) and pulse wave velocity (PWV). The objective of this prospective study was to assess the differences between arterial function in pregnant vs. non-pregnant women of the same age, and its changes throughout the gestation period. Materials and Methods. Between 2010–2019, 887 patients were enrolled into 2 groups: pregnant (N1 = 471) and non-pregnant (N2 = 416). Data about their anthropometric characteristics, arterial function (for group 1 in all three trimesters and 6 weeks post-partum), smoking status and physical activity were collected. Results. There were statistically significant differences (*p* < α, α = 0.05) between the two groups regarding the body mass index, brachial AIx, systolic, diastolic and central blood pressure, and pulse pressure values. In the first group, there was a decrease of both brachial AIx and PWV in the second and third trimester, followed by a post-partum increase; better outcomes were noticed in physically active women. Conclusions. Arterial function modifies during pregnancy and these alterations differ according to the trimester of gestation. Further research is needed to establish the cut-off values for this category. Pregnant women can have better outcomes through physical activity.

## 1. Introduction

Cardiovascular disease is associated with increased arterial stiffness and central aortic systolic blood pressure (SBPao) [1]. The first sign of vascular dysfunction and atherosclerosis is represented by arterial stiffening, which can be assessed through pulse wave velocity (PWV), a simple, non-invasive, and reliable parameter [2]. It is optimal to recognise subclinical atherosclerosis in asymptomatic populations and take action before clinical manifestations of cardiovascular diseases occur and to stratify the risk accordingly [3]. PWV has the advantage and potential to be utilised in the general population, and an enhancement in the capability of identifying high-risk patients would lead to improved stratification and more effective preventive therapy [4]. 

Arterial stiffness describes properties of the arterial vascular system such as distensibility, compliance, and elastic modulus [5]. It can be assessed at different sites, such as systemically (pulse wave analysis), regionally (PWV), or locally (relating a change in the area of an artery to distending pressure) [6]. PWV measures the distance travelled by the pulse wave over time, has the best predictive value for cardiovascular events and the simplicity of its measurement makes it a gold standard for assessing arterial stiffness in daily practice [7]. Among stiffness parameters, PWV is the most studied, and its predictive accuracy has been demonstrated in a number of studies [8]. 

Physiological pregnancy has a shifting effect on the cardiovascular system, which is maintained into the post-partum period [9]. At around five weeks of gestation, there is a systemic and renal vasodilatation; then, the systemic vascular resistance (SVR) decreases progressively (by 35–40%) until the middle of the second trimester, when it plateaus before beginning to increase late in the third trimester [10]. Endothelium-dependent vasodilatation and brachial artery diameter enhancement occur in healthy pregnancies [11]. Vasodilation causes a drop-in mean arterial pressure associated with increased cardiac output (from 20 to 50%) and plasma volume [12], due to the stroke volume increase of about 25% in the first trimester. This considerable increase in the cardiac output is one reason why pregnant women with pre-existing heart conditions can experience dramatic effects, especially later on. Arterial stiffness may be a measure of the degree of plasma volume expansion [13]. These changes are often hemodynamic, counter-regulatory and still maintain the basic vascular principles of keeping the new mean arterial pressure of pregnancy [14]; they are supposed to be caused by the attachment of the placenta to uterine walls, which induces the release of hormones and subsequent changes to maternal physiology [14]. Hormonal changes include an increased rate of release of vasodilators in the maternal female, such as prostaglandins and nitric oxide [15], and a decreased responsiveness of maternal vasculature to angiotensin II and norepinephrine [16]. 

Systemic vasodilation and physiologic anaemia lead to tachycardia (heart rate increases by 15–30% in the first trimester of pregnancy) and contribute to a further cardiac output augmentation [17]. Blood pressure slightly decreases early in pregnancy, due to incomplete compensation of cardiac output, and is more pronounced for the diastolic component, which returns to normal or even exceeds baseline values [18]. 

There are proofs regarding the contribution of maternal arterial dysfunction in pregnancy-specific conditions such as preeclampsia and intrauterine growth restriction [19]. Arterial stiffness is positively associated with hypertension [20], stroke [21], heart failure, and ischemic artery disease [22], with an increase in the risk of a first major cardiovascular event [23], which is the main cause of death in pregnant women in developed countries [24]. Arterial stiffness is an independent risk factor for atherosclerosis and a predictor of cardiovascular morbidity and mortality [25]. The Framingham Heart Study concluded that elevated stiffness correlates with an increased probability for a major cardiovascular event [26]. In the Rotterdam Study, it was observed that arterial stiffness independently predicts stroke and coronary heart disease in healthy normotensive patients [27]. In addition, the arterial stiffness is related to the foetus and neonatal outcomes as well. Similar to arterial stiffness, low birth weight is a predictor for cardiovascular morbidity and mortality [28].

The predictive value of PWV for cardiovascular diseases (CVD) has been studied and reported in the general population and in patients with different clinical conditions [2]. The information regarding the clinical significance of arterial distensibility assessment in young to middle age pregnant women is missing. Arterial function parameters could be useful tools to detect early modifications in the vascular function of pregnant patients before clinical manifestations and further complications occur in both mother and foetus. They could also serve for the assessment of therapeutic results. The lack of reference values in pregnant patients limits their applicability in clinical practice. 

In the general population, arterial stiffness is an early marker of cardiovascular disease and a predictor for mortality. During pregnancy, the entire cardiovascular system suffers modifications, including the arterial stiffness and compliance parameters. These alterations are more extensive and prolonged in preeclampsia patients. Arterial stiffness enhancement precedes the onset of pregnancy-related hypertensive disorders. Its parameters can be used as early risk detectors in normotensive, low-risk women who will develop preeclampsia. 

The current study aimed to compare arterial stiffness in pregnant and non-pregnant women and assess the differences. There are no reference values for gestational period and previous research was based on numbers for the general population. In all pregnant patients with pregnancy-related diseases, chronic conditions prior to gestation, or risk factors, or even healthy women, it is necessary to assess their arterial stiffness parameters as predictors for possible future events. It is mandatory to thoroughly study the arterial stiffness before, during and after gestation on large groups of subjects and to establish the exact cut-off values for this population.

## 2. Materials and Methods

### 2.1. Study Design

This prospective study was performed in patients admitted to the Obstetrics and Gynaecology Department of “Pius Brînzeu” Emergency Clinical County Hospital of Timisoara, Romania, during a 10-year period (between 2010–2019). The study was performed with the approval of the Ethics Committee of the hospital mentioned above (decision no. 54/20 April 2017) and it was conducted according to the established principles of the Declaration of Helsinki [29].

First, 1000 patients were included in the study. Patients with high blood pressure, cardiac arrhythmias, diabetes, obesity, hypercholesterolemia, chronic kidney disease, and systemic vasculitis, were excluded from the study. The final group consisted of 887 pregnant and non-pregnant healthy women, aged between 20–45 years, after they signed the informed consent.

**Figure 1:**
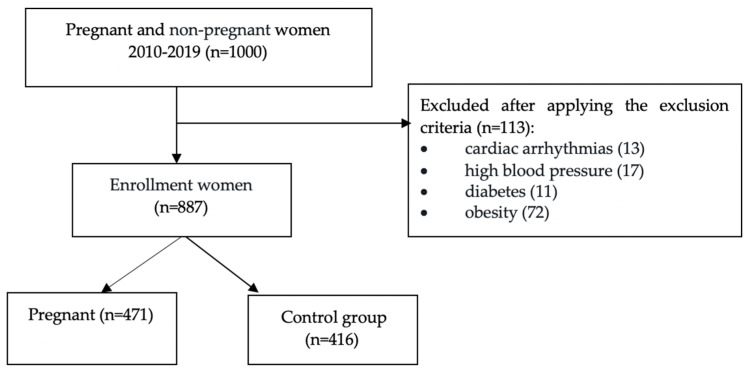
Flow chart of the study.

The patients were divided into two different groups: group 1—healthy primiparous pregnant patients (N1 = 471, 53.1%), without comorbidities or risk factors and group 2—control, healthy non-pregnant patients (N2 = 416, 46.9%). Figure 1 presents the flow chart of the study.

Personal data about lifestyle, smoking status, exercise, medical history (including family history), anthropometric features (such as age, height, weight, body mass index (BMI)) and results of arterial function assessments were collected. In group 1 (healthy pregnant), data about arterial functions were collected during four separate visits, in the first, second and third trimester of pregnancy, and 6 weeks post-delivery. In addition, data about the gestational period, the foetus weight and gender and delivery type (vaginal or caesarean) were added.

### 2.2. Paraclinical Evaluation

The pulse wave was analysed through an easy, non-invasive method and an accredited medical device: Arteriograph (TensioMed, Hungary). Each patient was prepared 24 h prior to the test, and the procedure was explained (according to the “patient info” document). For PWV determination, the protocol from the technical book [30] of the device was followed. 

The room/doctor’s office offered a proper environment (no noises, temperature of 22–25 ℃); the consultation bed was a horizontally fitted mattress with easy access to both arms. Patients who smoked, ate, drank energy drinks/coffee within <3 h prior to the assessment were rescheduled. The patient was laid down to rest for approximatively 10–20 min before the investigation. After the patient was set in the supine position, tight clothing was removed from forearms and arms. The circumference of both arms and the distance between the pubic symphysis and the sternum fork were measured. Personal information, anthropometric characteristics and anamnesis data were registered. The cuff recommended by the software (1, 2 or 3) was placed in the middle of the arm and the measurements were performed. The patient was not allowed to speak, sleep or move throughout the investigation, and remained in the supine position. If the investigation values appeared erroneous, or the patient was emotional or had tachycardia, the assessment was repeated 4 times (1–2 min intervals between measurements) on the same arm. The cuff was shifted to the contralateral arm (observing the size of the cuff according to the software recommendation) if the expected results were not obtained. If no results were obtained on the contralateral arm as well, manual measurements were performed, as allowed by the software. In the case of arrhythmias, the investigation was postponed until sinus rhythm was established and performed only in hemodynamic stability, with properly controlled values (according to the European Society of Cardiology guidelines) [30]. Patients who had pathological results were informed about their outcomes and referred to a cardiologist. The result of the determination was General Data Protection Regulation (GDPR) compliant [31].

According to the data from the literature, the reference value of PWV in adult women is 7.4 m/s [32]. Reference values are lower in young, healthy women (6.1 (4.6–7.5) m/s)), and gradually increase with age and blood pressure. Reference values for PWV [33] in the general population are presented in Table 1. 

The complex arterial function assessment included parameters such as systolic blood pressure (SBP), diastolic blood pressure (DBP), pulse pressure (PP), heart rate (HR), aortic SBP (SBPao), aortic pulse wave velocity (PWVao), and augmentation index (AIx). All measurements were performed with the same device, TensioMed Arteriograph, mentioned before.

### 2.3. Statistical Analysis

The database was created using the Microsoft Excel program. For testing the data distribution, the Kolmogorov–Smirnov normality test was applied. To see if the observed differences were statistically significant, the most suitable statistical tests for our variables, the *t* Student test, Mann–Whitney test, ANOVA unifactorial test and Friedman test were used. For the entire study, α = 0.05 was considered as the confidence level. For descriptive statistics, the central tendency and dispersion indicators were calculated, and the main results were plotted. The statistical analysis was performed using the SPSSv17 and the Microsoft Excel programs.

## 3. Results

For the qualitative dichotomous variables, frequency tables were used. The mean values for basic characteristics and hemodynamic parameters in both groups are presented in Table 2.

To determine if there are any significant differences between both groups and between subjects who performed physical activity and who did not, several statistical tests were used. The Kolmogorov–Smirnov normality test was applied to see the data distribution for the numerical variables, and a normal distribution for all data was found (*p* < 0.05); thus, parametrical tests were valid. For double-checking, one parametrical test (the Student t unpaired test) and a nonparametric test (the Mann–Whitney test) for age, body mass index (BMI), brachial AIx, PWVao, SBP, DBP, PP (which is the difference between systolic and diastolic blood pressure), SBPao and HR values were combined. Statistically significant differences (*p* < 0.05) were obtained for BMI, brachial AIx, PWVao, DBP, PP and SBPao values. All the results are presented in Table 3.

In pregnant women, brachial AIx decreased progressively during pregnancy and increased again in post-partum (the mean value in the first trimester was −47.41% ± SD, in the second trimester it was −51.93% ± SD, and in the third it was −58.40% ± SD; in post-partum, it increased to −45.57% ± SD). PWVAo started to decline in the second trimester and continued to decline in the third trimester; in post-partum, it was increased compared to the first trimester (mean PWVAo in the first trimester was 6.78 m/s ± SD, in the second trimester 5.99 m/s ± SD and in the last trimester 5.93 m/s ± SD; in post-partum it increased to 7.17 m/s ± SD) (Table 4).

**Figure 2:**
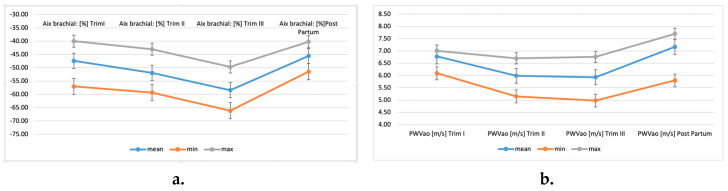
The brachial AIx (**a**) and PWVao progressions (**b**), in group 1 (healthy pregnant patients, N1 = 471).

In order to see if there are significant differences in the four tested time points, the ANOVA unifactorial test and Friedman test were applied, resulting in extremely significant differences (*p* < 0.001) between the brachial AIx (%) and PWVao values during the study period. Brachial AIx % decreased until the third trimester, when it started to increase again until the post-partum stage (Figure 2a). PWVao decreased in the second trimester, it was maintained in the last trimester, and increased again in post-partum, even more, compared to baseline (first trimester) (Figure 2b).

The mean values for the gestational period (39.34 weeks ± SD), APGAR score (9.22 ± SD) and foetus weight (3468.97 g ± SD) are presented in Table 5. The lifestyle, habits, pregnancy and delivery outcomes, as well as foetus gender, for both groups are presented in Table 6. 

To see if physical activity can modify the outcomes, both the Student *t* unpaired test and Mann–Whitney test were applied, for each group separately. Significant differences were obtained (*p* < 0.05) for BMI, brachial AIx, PWVao, SBP, DBP, PP, SBPao and HR values. In group 1, women who regularly exercised (33.97%) were compared to women who did not exercise (66.03%). We also compared them in all 4-time moments: first, second, third trimester, and in post-partum. Patients who are physically active had statistically significant better outcomes (*p* < 0.05) for delivery, anthropometric features, hemodynamic status and arterial functions.

## 4. Discussion

PWV is an independent predictor of future CVD in women with high blood pressure [22], stroke [34], coronary artery disease [35], diabetes [36], and end-stage renal disease [37]. Reference values for pregnant women need to be established for better assessment and early detection. In the current study, healthy pregnant women had statistically significantly different values compared to healthy non-pregnant women; thus, larger prospective studies are necessary, to establish reference values for arterial function parameters for this category.

Lynch et al. highlighted the critical role of communication between physicians and pharmacists for the best outcomes of pregnancy [38]. The inter-professional team is an essential part of the healthcare system, and the need for balanced coordination of care only expands in managing the pregnant patient through a safe pregnancy for the mother and child [39].

Numerous studies have demonstrated the prognostic significance of PWVao measurement as an indicator of subclinical organ damage, both in the general population and patients with increased cardiovascular risk, especially patients with hypertension, diabetes, or chronic renal failure. Evidence suggests that aortic stiffness may precede and contribute initially to the development of hypertension [40].

In a healthy pregnancy, there is a significant increase in uterine blood flow, remodelling of the spiral arteries [41], more nitric oxide, generated from the endothelium, and enhanced overall vasodilation of the uterine vessels [42]. Women who develop preeclampsia have a decreased flow-mediated dilatation in the first and second trimesters compared to healthy pregnancies [43]. The parameters of arterial stiffness are significantly different in preeclamptic women compared to normotensive pregnancies [19] but there are no cut-off values in the literature for pregnant women.

Considering the literature data, we hypothesised that pregnant women would have a different pattern of arterial function compared to non-pregnant women. This field is yet to be explored. Some studies concluded that in physiological pregnancy, there is a significant decrease in unadjusted aortic PWV from pre-conception to the second trimester [19]. In the present research an interesting progression of the arterial markers was observed: starting from baseline (at enrolment), brachial AIx % decreased until the third trimester, and then started to increase again until the post-partum stage. PWVao decreased in the second trimester, and was maintained in the last trimester, and increased again in post-partum, even more compared to baseline (first trimester). This specific pattern should be studied in larger cohorts in the future. 

Fujime et al. observed that central aortic SBP (cSBP) and AIx significantly declined during pregnancy, reaching lowest values in mid-pregnancy and rising towards term, especially the heart rate-corrected AIx (AIx-75). cSBP and AIx-75 were significantly increased in healthy pregnant women older than 35 years.

Beck et al. demonstrated that resistance and endurance exercise alone effectively reduce peripheral arterial stiffness, central blood pressure, AIx, and myocardial oxygen demand in young pre-hypertensive subjects [44]. Further on, it was demonstrated that exercise training programs improve hemodynamic parameters such as SBP, PP, SBPao, and may delay arterial ageing [45] in hypertensive populations [46]. Regarding pregnant subjects, Beetham et al. emphasized in their systematic review that sustained exercise during the third trimester appears to be safe for most healthy pregnant women [47]. This is concordant to the results of our study. In addition, physical activity improved the well-being of the previously inactive/underactive patients. Healthy pregnant women had significant benefits from regular exercise compared to sedentary healthy pregnant women (*p* < 0.05) in terms of delivery, anthropometric features, hemodynamic status and arterial functions. 

Numerous pharmacological [48,49] and non-pharmacological [50] solutions are able to reduce PWV and could offer an alternative for patients at high risk of CVD [51,52]. Nonetheless, further studies are necessary to confirm whether PWV reduction by this approach can directly prevent CVD in pregnant women. It is necessary to work with appropriately adjusted parameters alongside raw data. There is little information in the medical literature regarding the effects of therapy on arterial function.

This study offers insights regarding PWV analysis and additional information about arterial stiffness in pregnant women versus conventional brachial blood pressure measurements [53]. In addition, the arterial pattern in pregnant women is different from non-pregnant women. This could be a potential base for further research regarding the importance of PWV study for the assessment, management and prediction of pregnancy-related cardiovascular events. Future perspectives include establishing exact cut-off values of arterial stiffness for healthy pregnant women and demonstrating the objective benefits of physical therapy during pregnancy in larger groups.

## 5. Conclusions

The results obtained in this study proved that arterial function is modified during pregnancy and these alterations differ according to the trimester of gestation; there was a decrease of both Aix brachial and PWV in the second and third trimester, followed by a post-partum increase. In addition, women who regularly exercised had ameliorated anthropometric features, hemodynamic status and delivery outcomes. Due to these specific changes that occur in normal healthy pregnancies, further studies are necessary to establish cut-off values for this category of patients, in order to detect early changes in arterial stiffness parameters. Arterial stiffness assessment represents a useful tool in the early diagnosis of pregnancies at risk and can lead to better routine care and better outcomes for these patients, through early detection and physical activity.

## Figures and Tables

**Table 1:** PWV reference values (m/s) in the general population according to the age category.

Age Category (Years)	Mean (+2 SD) *	Median (10–90 pc) **
<30 years	6.2 (4.7–7.6)	6.1 (5.3–7.1)
30–39 years	6.5 (3.8–9.2)	6.4 (5.2–8.0)
40–49	7.2 (4.6–9.8)	6.9 (5.9–8.6)
50–59	8.3 (4.5–12.1)	8.1 (6.3–10.0)
60–69	10.3 (5.5–15.0)	9.7 (7.9–13.1)
≥70	10.9 (5.5–16.3)	10.6 (8.0–14.6)

* SD, standard deviation; ** 10 pc, the upper limit of the 10 th percentile; 90 pc, the lower limit of the 90 th percentile.

**Table 2:** Anthropometric, haemodynamic and arterial stiffness characteristics of both groups (group 1, N1 = 471—healthy pregnant patients; group 2, N2 = 416—control group).

Statistics	Age	BMI (Kg/m^2^)	SBP (mmHg)	DBP (mmHg)	PP (mmHg)	SBPao (mmHg)	HR (1/min)	Brachial AIx (%)	PWVao (m/s)
Mean	30.15	21.43	122.30	72.62	49.68	115.21	72.28	−47.41	6.78
	29.61	21.43	122.59	74.72	47.87	109.16	71.57	−49.50	7.43
Standard error	0.24	0.10	0.49	0.28	0.30	0.14	0.16	0.20	0.01
	0.22	0.09	0.42	0.30	0.28	0.55	0.48	0.79	0.02
Median	27	20.76	130	75	54	116	73	−47.47	6.81
	31	21.36	122	75	48	110	71	−50.7	7.4
Mode	27	19.96	130	75	55	116	73	−52.6	6.78
	32	22.58	130	80	40	119	68	−34.5	7.4
Standard deviation	5.22	2.27	10.72	6.06	6.51	3.11	3.49	4.36	0.16
	4.39	1.86	8.64	6.09	5.69	11.22	9.75	16.20	0.49
Sample variance	27.24	5.14	114.98	36.70	42.35	9.66	12.17	19.02	0.02
	19.26	3.47	74.60	37.12	32.33	125.80	95.14	262.60	0.24
Kurtosis	−0.79	−0.87	0.19	−0.06	−0.20	0.81	0.11	−1.25	6.17
	0.30	−0.81	−0.94	−0.58	−0.86	0.04	0.03	0.45	7.95
Skewness	0.77	0.50	−1.15	−0.05	−0.94	−0.95	−0.68	−0.04	−2.18
	0.11	0.19	0.04	−0.05	0.28	0.04	0.45	0.41	2.26
Range	25	9.65	36	25	26	13	16	17	0.92
	25	7.52	34	30	20	56	53	101.6	3
Minimum	20	17.30	94	60	29	107	63	−57	6.09
	20	18.20	108	62	40	82	52	−85.9	6.7
Maximum	45	26.95	130	85	55	120	79	−40	7.01
	45	25.71	142	92	60	138	105	15.7	9.7

Data for the group 1 were highlighted in grey.

**Table 3:** The primary descriptive statistics, the *p* values obtained using both Student *t* unpaired test and the Mann–Whitney test, applied on the numerical variables for both groups (N1 = 471, N2 = 416).

Statistics → Variables ↓		Group	Mean	Standard Deviation	Std. Error Mean	*p* Value *t*-Test	*p* Value Mann–Whitney
Age	Years	1	30.15	5.2	0.241	0.096	0.607
		2	29.61	4.39	0.22		
BMI	Kg/m^2^	1	21.42	2.2	0.1045	<0.001 *	0.034 *
		2	21.43	1.86	0.09		
Brachial AIx	%	1	−47.41	4.36	0.20	0.011*	0.012 *
		2	−49.50	16.20	0.79		
PWVao	m/s	1	6.77	0.157	0.007	<0.001 *	<0.001 *
		2	7.43	0.49	0.023		
SBP	(mm Hg)	1	122.30	10.723	0.494	<0.001 *	<0.001 *
		2	122.59	8.64	0.42		
DBP		1	72.62	6.058	0.279	<0.001 *	<0.001 *
		2	74.72	6.09	0.3		
PP		1	49.68	6.507	0.300	<0.001 *	<0.001 *
		2	47.87	5.69	0.28		
SBPao		1	115.21	3.108	0.143	<0.001 *	<0.001 *
		2	109.16	11.22	0.55		
HR	1/min	1	72.28	3.488	0.161	<0.001 *	<0.001 *
		2	71.57	9.75	0.48		

* Statistically significant differences (*p* < 0.05).

**Table 4:** The central tendency and dispersion indicators calculated on arterial stiffness characteristics for group 1 (healthy pregnant patients, N1 = 471).

Statistics	Brachial AIx (%)	PWVao (m/s)	Brachial AIx (%)	PWVao (m/s)	Brachial AIx (%)	PWVao (m/s)	Brachial AIx (%)	PWVao (m/s)
	1st trimester		2nd trimester		3rd trimester		Post-partum	
Mean	−47.41	6.78	−51.93	5.99	−58.40	5.93	−45.57	7.17
Standard error	0.20	0.01	0.11	0.02	0.22	0.02	0.16	0.02
Median	−47.47	6.81	−52.18	6.1	−58.45	6.07	−45.16	7.35
Mode	−52.6	6.78	−52.6	6.1	−52.6	6.14	−45.16	7.39
Standard deviation	4.36	0.16	2.43	0.51	4.73	0.53	3.54	0.43
Sample variance	19.02	0.02	5.89	0.26	22.37	0.28	12.51	0.18
Kurtosis	−1.25	6.17	1.86	−1.65	−1.41	−1.18	−1.38	4.64
Skewness	−0.04	−2.18	0.10	−0.04	0.13	−0.31	−0.21	−2.43
Range	17	0.92	16.3	1.55	16.46	1.78	11.28	1.9
Minimum	−57	6.09	−59.3	5.15	−66.15	4.98	−51.5	5.8
Maximum	−40	7.01	−43	6.7	−49.69	6.76	−40.22	7.7

**Table 5:** Descriptive statistics of obstetrical characteristics for group 1 (healthy pregnant patients, N1 = 471).

Statistics	Gestational Period (Weeks)	APGAR Score at 1 min	Foetus Weight (g)
Mean	39.34	9.22	3468.97
Standard error	0.05	0.04	6.86
Median	39	9	3500
Mode	40	10	3500
Standard deviation	1.05	0.95	148.84
Sample variance	1.09	0.90	22,154.52
Kurtosis	0.97	11.92	−0.09
Skewness	−0.30	−2.17	0.18
Range	7	9	820
Minimum	35	1	3100
Maximum	42	10	3920

**Table 6:** The frequency table according on the qualitative characteristics of both groups.

Variables	Yes/Yes/Girl/Natural		No/No/Boy/C-Section	
	Number	%	Number	%
**Group 1 (N1 = 471)**				
Exercise	160	33.97	311	66.03
Smoker	194	41.19	277	58.81
Baby gender	204	43.31	267	56.69
Delivery type	233	49.47	238	50.53
**Group 2 (Control Group, N2 = 416)**				
Exercise	191	45.91	225	54.09
Smoker	160	38.46	256	61.54
